# Perspectives of healthcare professionals and older patients on shared decision-making for treatment escalation planning in the acute hospital setting: a systematic review and qualitative thematic synthesis

**DOI:** 10.1016/j.eclinm.2023.102144

**Published:** 2023-08-10

**Authors:** Bronwen E. Warner, Adam Lound, Kate Grailey, Cecilia Vindrola-Padros, Mary Wells, Stephen J. Brett

**Affiliations:** a Division of Anaesthetics, Pain Management and Intensive Care, Department of Surgery and Cancer, Imperial College London, UK; b Patient Experience Research Centre, School of Public Health, Imperial College London, London, UK; c Centre for Health Policy, Institute for Global Health Innovation, Department of Surgery and Cancer, Imperial College London, UK; d Department of Targeted Intervention, University College London (UCL), London, UK; e Department of Surgery and Cancer, Imperial College London, UK; f Imperial College Healthcare NHS Trust, London, UK; g Department of Intensive Care Medicine, Imperial College Healthcare NHS Trust London, London, UK

**Keywords:** Treatment escalation, Shared decision-making, Older people, Triage decisions, Qualitative research

## Abstract

**Background:**

Shared Decision-Making (SDM) between patients and clinicians is increasingly considered important. Treament Escalation Plans (TEP) are individualised documents outlining life-saving interventions to be considered in the event of clinical deterioration. SDM can inform subjective goals of care in TEP but it remains unclear how much it is considered beneficial by patients and clinicians. We aimed to synthesise the existing knowledge of clinician and older patient (generally aged ≥65 years) perspectives on patient involvement in TEP in the acute setting.

**Methods:**

Systematic database search was performed in MEDLINE, EMBASE, PsycInfo and CINAHL databases as well as grey literature from database inception to June 8, 2023, using the Sample (older patients, clinicians, acute setting; studies relating to patients whose main diagnosis was cancer or single organ failure were excluded as these conditions may have specific TEP considerations), Phenomenon of Interest (Treatment Escalation Planning), Design (any including interview, observational, survey), Evaluation (Shared Decision-Making), Research type (qualitative, quantitative, mixed methods) tool. Primary data (published participant quotations, field notes, survey results) and descriptive author comments were extracted and qualitative thematic synthesis was performed to generate analytic themes. Quality assessment was made using the Critical Appraisal Skills Programme and Mixed Methods Appraisal Tools. The GRADE-CERQual (Grading of Recommendations Assessment, Development and Evaluation–Confidence in the Evidence from Reviews of Qualitative research) approach was used to assess overall confidence in each thematic finding according to methodology, coherence, adequacy and relevance of the contributing studies. The study protocol was registered on PROSPERO, CRD42022361593.

**Findings:**

Following duplicate exclusion there were 1916 studies screened and ultimately 13 studies were included, all from European and North American settings. Clinician-orientated themes were: treatment escalation is a medical decision (high confidence); clinicians want the best for their patients amidst uncertainty (high confidence); involving patients and families in decisions is not always meaningful and can involve conflict (high confidence); treatment escalation planning exists within the clinical environment, organisation and society (moderate confidence). Patient-orientated themes were: patients’ relationships with Treatment Escalation Planning are complex (low confidence); interactions with doctors are important but communication is not always easy (moderate confidence); patients are highly aware of their families when considering TEP (moderate confidence).

**Interpretation:**

Based on current evidence, TEP decisions appear dominated by clinicians' perspectives, motivated by achieving the best for patients and challenged by complex decisions, communication and environmental factors; older patients’ perspectives have seldom been explored, but their input on decisions may be modest. Presenting the context and challenge of SDM during professional education may allow reflection and a more nuanced approach. Future research should seek to understand what approach to TEP decision-making patients and clinicians consider to be optimum in the acute setting so that a mutually acceptable standard can be defined in policy.

**Funding:**

HCA International and the 10.13039/501100013342NIHR Imperial Biomedical Research Centre.

**Research in context**

**Evidence before this study**

Shared Decision-Making (SDM) is increasingly expected in healthcare, including in Treatment Escalation Planning (TEP), but it is not clear how this is perceived by patients and clinicians. We scoped existing evidence on SDM in TEP in a preliminary search of MEDLINE, EMBASE, PsycInfo and CINAHL databases as well as grey literature from database inception to SEPT 2022 with no restriction by language. We searched terms relating to the emergency medical setting, Treatment Escalation Planning and Shared Decision-Making and identified a number of studies exploring TEP decision-making. An evidence synthesis of processes, barriers and facilitators related to Do Not Attempt Cardiopulmonary Resuscitation decision-making and implementation was published in 2016, but this focussed on the DNACPR decision rather than broader TEP and did not specifically examine shared decision-making.

**Added value of this study**

This comprehensive synthesis is the first we are aware of exploring perceptions of SDM around TEP in the acute hospital setting. We find with high confidence that treatment escalation is considered by clinicians to be a medical decision, clinicians want the best for their patients amidst uncertainty, and clinicians find that involving patients and families in decisions is not always meaningful and can involve conflict. With moderate confidence, we find that treatment escalation planning exists within the clinical environment, organisation and society, patients find interactions with doctors important but communication is not always easy, and patients are highly aware of their families when considering TEP. We also find with low confidence that patients’ relationships with Treatment Escalation Planning are complex.

**Implications of all the available evidence**

Contrary to policy and sociocultural expectations of SDM in Western settings, TEP decisions appear dominated by clinicians' perspectives, motivated by achieving the best for patients and challenged by complex decisions, communication and environmental factors; older patients’ perspectives remain unclear, but their input on decisions may be modest. Future research should seek to understand what TEP decision-making approach patients and clinicians consider to be optimum in the acute setting.

## Introduction

Population distribution is shifting worldwide towards older age.^1^ Frailty, multimorbidity and disability increase with age.^2^^,^^3^ It is challenging to prognosticate survival from severe illness and future quality of life for older people.^4^^,^^5^

With expanding scope for life-saving medical treatments, decision-making around *appropriateness* of such intervention is increasingly complex and pertinent. Following Do Not Attempt Cardiopulmonary Resuscitation (DNACPR) orders in the 1970s,^6^ Treatment Escalation Plans (TEPs) were conceptualised in the 1990s^7^ amidst a cultural shift towards greater patient involvement in decision-making.^8^ They are now used in several advanced health systems.^9^, ^10^, ^11^, ^12^

TEPs outline interventions to be considered in clinical deterioration. They are designed to reflect individual patient preferences and clinician expertise.^13^ TEP conversations are immediately relevant for emergency medical inpatients where chance of deterioration is higher.^13^, ^14^, ^15^, ^16^ The high burden of acute patients during COVID-19 demonstrated challenge and importance of inpatient TEP^17^ with particular debate about escalation of care for older people.^18^ Optimising TEP decision-making approaches in the acute medical setting is a research and policy focus.^12^^,^^19^

Shared decision-making (SDM) between expert clinician and informed patient is a collaborative process where patient and healthcare professional make a joint decision about immediate or future care.^20^ It is increasingly expected in ‘western’ societies^20^, ^21^, ^22^ and less established worldwide.^23^^,^^24^ SDM reflects a cultural move away from paternalism towards greater patient empowerment.^25^ Models can encompass a spectrum of patient involvement,^26^^,^^27^ but usually involve presenting more than one management option and prioritising individualised communication.^28^ SDM is an area of active research^29^, ^30^, ^31^ and can increase patient trust, understanding and satisfaction.^32^ Historically studied in primary care,^33^ it is more recently discussed in the emergency setting,^34^ where time pressure, complexity and acuity can challenge SDM.^35^, ^36^, ^37^ In TEP, SDM can inform subjective goals of care.^38^ This perspective is endorsed by recent high-profile UK legal rulings mandating that patients or next of kin be involved in CPR decisions^39^^,^^40^ and in guidance from professional bodies.^16^

Despite policy and ideological support of SDM in acute TEP, it remains unclear how much it is considered feasible, meaningful or desirable by patients and clinicians. This review will synthesise existing knowledge of clinician and older patient perspectives on patient involvement in acute setting TEP, with potential implications for researchers and policy makers.

The aim of this study was to understand i) what are the experiences and perspectives of clinicians making TEP decisions with older patients in the acute medical setting; and ii) what are the experiences and perspectives of older patients regarding TEP decision discussions with clinicians in the acute medical setting.

## Methods

This is a synthesis of data from primary studies. In keeping with the systematic review approach, explicit and reproducible methodology is used comprising systematic search for relevant studies, assessment of bias and systematic synthesis of the studies included.^41^ As the review question seeks to understand perspectives and meaning, a qualitative evidence synthesis approach was used^42^ following Cochrane guidance^43^ and the ENTREQ checklist.^44^

The protocol was registered on PROSPERO (reference number: CRD42022361593). No ethics committee approval was required for this synthesis.

### Search strategy and selection criteria

Primary studies were included regardless of methodology. The search strategy was developed in consultation with a librarian expert in medical research literature and presented using the Sample, Phenomenon of Interest, Design, Evaluation, Research type (SPiDER) tool^45^ (Table 1).

**Table 1:** Systematic search strategy.

SPiDER tool	Topic of interest	Definition	Search terms
**Sample**	Older patients with capacity, Clinicians Acute medical setting	This synthesis focuses on older patients. We have generally defined ‘older’ as patients aged ≥65 years as a pragmatic accepted threshold.^46^ Where age of participants was not specified, we excluded studies specifically including only patients <65 years but included studies which were largely comprised of older patients. We did not use a strict age cut-off, as the purpose of the review was a thematic synthesis and it was felt that findings would remain relevant to our research questions. Studies including only patients with single organ failure or cancer were excluded, as these conditions may have specific treatment escalation planning considerations. We also excluded studies where the decision is primarily regarding targeted interventions for a specific condition or a decision about undergoing surgery. We did not include studies only looking at surrogate perspectives, but if a study included surrogate as well as patient perspectives it remained eligible. The setting contextualising the TEP decision. For example, participants were included who were working in the acute setting or were a current inpatient, but also those where the scenario considered involved TEP decisions being made in an acute inpatient setting. Decisions made regarding a community setting (e.g. outpatients or residential home) were excluded.	Emergency MESH e.g. (exp emergency medicine/or exp emergency treatment/or exp emergency ward/or emergency health service/or exp emergency patient/or exp emergency care/or exp hospital emergency service/or exp evidence based emergency medicine/or exp emergency physician/) Emergenc∗ Acute medic∗ Acute depart∗ Acute service Acute care
**Phenomenon of interest**	Treatment Escalation Planning	TEP was defined as ‘recommendations for a person's clinical care in a future emergency in which they do not have capacity to make or express choices'.^47^ Terms relating to TEP rather than only CPR were used as we aimed to capture decisions considering a range of treatment options.	Treatment escalat∗ Recommended summary plan for emergency care and treatment Ceiling of care Ceiling of treatment Limit treatment∗ Treatment limit∗ life sustaining treatment MESH life sustaining treatment Physician order∗ for life sustaining treatment Emergency care and treatment plan Emergency care and treatment plan∗ Emergency care treatment plan∗ treatment escalation limitation plan No escalation of treatment Ward based ceiling Full escalat∗
**Design**	Any design including survey, interview, focus group, observational		
**Evaluation**	Shared decision-making	Studies were only included if they explored perceptions on decision-making between a clinician and a patient. Therefore, studies exploring how patients or clinicians made these decisions independently were not included.	Decision making MESH (clinical decision making, shared decision-making, medical decision making) Doctor patient relationship MESH Decision∗ physician attitude∗ doctor patient relation∗ Physician patient relation∗ Doctor patient communication Physician patient communication interpersonal communication Attitude of Health Personnel
**Research type**	Qualitative, quantitative or mixed methods		

SPiDER = Sample, phenomenon of interest, design, evaluation, research type; TEP = Treatment escalation plan; CPR = Cardiopulmonary resuscitation; MESH = Medical subject heading.

MEDLINE, EMBASE, PsycInfo and CINAHL databases were searched from database inception to SEP27, 2022 and the search updated JUN08, 2023. Search strategies were developed for each database, in keeping with systematic search methodology^48^ (Appendix). No limits were applied on publication date, but only articles in English were included. A grey literature search was conducted using Open Grey and Trip. Reference lists of review articles identified in the primary search were hand-searched to identify additional articles for inclusion.

BW and AL independently assessed titles and abstracts for initial eligibility, followed by full text review of potentially relevant papers. The systematic review management software Covidence (Veritas Health Innovation, Melbourne, Australia, available at www.covidence.org) was used to support double reviewer involvement. Any initial disagreement was resolved through discussion which prompted closer review and subsequent agreement.

### Data analysis

#### Data extraction

Descriptive information of included papers was gathered by BW: year of publication, country, research question/aim, whether the data was primary or secondary, healthcare setting, health conditions, participant type, number of participants, age of patients, study design/data collection methods, recruitment, analysis methods, theoretical framework (Table 2).

**Table 2:** Descriptive analysis of included studies.

Study	Research question/aim	Primary or secondary	Healthcare setting	Health conditions	Participant type (patients/clinicians)	Number of participants	Age (if patient)∗	Study design, data collection methods	Recruitment	Analysis methods	Stated theoretical framework
Eli 2020, England^49^	To examine secondary care consultant clinicians' experiences of conducting conversations about treatment escalation with patients and their relatives, using the Recommended Summary Plan for Emergency Care and Treatment (ReSPECT) process.	Primary	Two National Health Service hospitals	Medicine and surgery	Medical and surgical consultants from 10 specialties, observed in 14 wards	15	Not specified	Ethnographic Observation and interview	Purposive sampling for a range of views and diversity of clinical areas	Thematic analysis	
Eli 2021, England^50^	To understand how ReSPECT conversations unfold in practice, examining why, when and how clinicians enact the ReSPECT process in hospital settings.	Primary	Six acute NHS trusts	Medicine and surgery	Consultant, middle grade and junior doctors	49 ReSPECT conversations observed, conducted by 34 clinicians. 31 interviews	32/49 participants were aged 80+ years	Ethnographic Observation and interview	Not specified	Inductive thematic analysis	
Eli 2022a, England^51^	To develop an ethnographic account of how and why clinicians defer and avoid ECTP conversations and how they rationalise these decisions as they happen	Secondary of Eli 2021	Six acute NHS trusts	Medicine and surgery	Consultant, middle grade and junior doctors (observed and interviewed), patients (observed)	34 doctors observed, 32 interviewed; 6 cases selected for in-depth analysis	Not specified	Ethnographic Observation and interviews	Not specified	“thick description of each case"	
Eli 2022b, England^52^	Why are some ReSPECT conversations left incomplete?	Secondary of Eli 2021	Six acute NHS trusts	Medicine and surgery	Consultant, middle grade and junior doctors (observed and interviewed), patients (observed)	6 incomplete conversations	n/a	Case study approach Ethnographic Observation and interviews	Not specified	Thematic analysis	critical realist
Escher 2021, Switzerland^53^	To determine which factors influence physicians' admission decisions in situations of potentially non-beneficial intensive care	Secondary analysis of study examining the triage process	Tertiary care centre	Internal medicine	ICU physicians and internists routinely involved in ICU admission decisions	24	n/a	in-depth interviews	Convenience and snowball	inductive approach to thematic content analysis	
Fassier 2016, France^54^	To explore physician's perceptions of and attitudes towards end-of-life decisions for elderly critically ill patients at the ED-ICU interface	Primary	Hospital	ED, short stay unit, step down unit, medical ICU. medicosurgical ICU. 5 multimorbidity cases highlighted	Clinicians	20 observed and interviewed; 4 interviewed only	selected cases aged 71–90 yrs	Ethnographic Observation and interviews	Purposive (sex, seniority, specialty) and snowball	Thematic analysis	
Jenkins 2015, USA^55^	Under what conditions do internal medicine residents limit or terminate treatment without respecting patient wishes?	Secondary analysis of study examining hierarchy within the medical profession	Community hospital	General medicine and some ICU	Internal medicine residents and attendings	97 observation sessions of approximately 45 clinicians	>80 yrs	Ethnographic Observation and interaction		Coding with reflexivity, theory generation (analysis approach not named)	
Rodriquez 2006, USA^56^	To explore patients' beliefs about control of their end of life health and health care	Primary	outpatient primary care clinic	not specified	Patients	30	60–81 years	semi-structured interviews	convenience sampling in anticipation of routine visit	constant comparative method	Grounded theory
Shah 2017, Canada^57^	To observe how residents are engaging with goals of care discussions with patients and identify thematic patterns that inhibited and promote discussion about goals of care	Primary	Academic teaching hospital	Internal medicine, family medicine, emergency medicine, general surgery	Patients (observed and interviewed) and clinicians (observed and survey)	15 resident-patient encounters or which 12 included a goals of care discussion	>65 yrs	audio-recording of encounter between patient and resident (recorded by resident, not observed); semi-structured interview with patient; survey for resident doctors		Qualitative content analysis with minimal theoretical interpretation; secondary analysis looking at how often residents addressed guideline-recommended goals of care discussions; statistical descriptive analysis of survey	
Tuesen 2022a, Denmark^58^	To explore patients' and physicians' perspectives on a decision-making conversation for life-sustaining treatment based on the Danish model of the POLST form	Primary	Primary and secondary care, nursing home	Serious illness and/or frailty	Patients and clinicians	6 patients and 5 clinicians	40–85+	semi-structured interviews	Purposive and convenience	Thematic analysis	
Tuesen 2022b, Denmark^19^	To develop and pilot test a Danish POLST form to ensure that patients' preference for levels of life-sustaining treatment are known and documented	Primary	hospital wards, general practitioners' clinics, home care and nursing homes	Serious illness and/or frailty	Patients, family members, clinicians and nurses	45 questionnaire and 14 interviews	18+	Questionnaires and in-depth interviews	Purposive and convenience	Descriptive statistics. Systematic text condensation	
Walzl 2019, Scotland^59^	To determine the factors that influence ceiling of treatment institution in the ED	Primary	Emergency department	Not specified	Clinicians (ED consultants)	15	n/a	semi-structured interviews	Convenience sampling	Thematic analysis	
You 2015, Canada^60^	To determine, from the perspective of hospital based clinicians 1) barriers impeding communication and decision making about goals of care with seriously ill hospitalised patents and their families and 2) their own willingness and the acceptability for other clinicians to engage in this process	Primary	Hospital–Medical teaching unit	General internal medicine	Nurses, internal medicine residents and staff physicians	1256	n/a	Survey paper- and web based- self-administered questionnaire	distributed locally by site investigators at each of 13 sites	statistical analysis	

ReSPECT = Recommended Summary plan for emergency care and treatment; ED = Emergency department; ICU = Intensive care unit; POLST = Physician orders for life sustaining treatment.

What constitutes ‘data’ for thematic synthesis has not been consistently defined.^46^^,^^47^ In this synthesis, the majority of evidence considered is primary data (participant interview quotations, observer field-notes, survey results) which were retrieved from anywhere in the manuscripts, most often the results section. Descriptive but not highly interpretive author comments were also included.

#### Assessing methodological limitations of included studies

Cochrane guidance informed selection of the quality assessment tool.^61^ The assessments were performed by BW and cross-checked by AL: qualitative studies using the Critical Appraisal Skills Programme (CASP) tool^62^; quantitative and mixed methods studies using the Mixed Methods Appraisal Tool.^63^ Quality assessment informed confidence in review findings but not study exclusion. In keeping with guidance,^61^, ^62^, ^63^ we have described study quality but not assigned a score.

#### Data analysis and synthesis

A convergent integrated approach facilitated analysis of different research types. Quantitative data was ‘qualitized’^64^ by labelling quantitative data with descriptive codes which were processed together with descriptive codes from the qualitative studies. All data were analysed qualitatively using thematic synthesis.^46^^,^^47^

Thematic synthesis is an accessible and recognised method for synthesising qualitative research which demonstrates an ‘audit trail’ to improve transparency.^42^^,^^61^ Each paper was read multiple times to gain familiarisation. Data were first coded inductively ‘line-by-line’ with no formal preconceptions of potential analytic themes. All studies were coded in this way prior to moving on to the next level of analysis to remain open to new findings. Next, codes were categorised as patient- or clinician- orientated. Codes were then grouped into descriptive themes; this process involved multiple rearrangements and sometimes amalgamation of codes. Concurrently, the original papers were summarised and reviewed iteratively to ensure that the descriptions remained ‘close’ to the primary studies. Finally, the analytical themes which constitute our findings were generated as interpretive constructs.^65^ Subthemes were subsequently developed within the main themes to articulate clusters of concepts within the main themes. Analysis was supported by computer assisted qualitative data analysis software NVivo (NVivo qualitative data analysis software, QSR International Pty Ltd, release 1.7.1).

A schematic was devised using synthesis findings to demonstrate existing knowledge and priorities for future research.

#### Assessing confidence in the review findings

The GRADE-CERQual (Grading of Recommendations Assessment, Development and Evaluation–Confidence in the Evidence from Reviews of Qualitative research) approach was performed by BW and independently cross-checked by AL to assess confidence in each finding according to methodology,^66^ coherence,^67^ adequacy,^68^ relevance^69^ and overall confidence.^65^ Confidence is judged as high, moderate, low, or very low; all findings start as high confidence and are graded down if there are important concerns regarding any of the GRADE-CERQual components.

#### Reflexivity

BW, AL, KG, MW and SJB have clinical experience (BW, SJB and KG as doctors in acute care, AL as a physiotherapist with expertise in chronic care, MW in cancer nursing) and recognise that this may influence their approach, for example by being more ready to appreciate findings based on perspectives aligning with their own. BW is a PhD candidate exploring treatment escalation decision-making. All authors have prior experience of qualitative methods research in the healthcare setting. A reflexive position was maintained throughout the analysis by BW through use of a reflexive diary and discussion with co-authors.

### Role of the funding source

The funders of the study had no role in study design, data collection, data analysis, data interpretation, or writing of the report.

All authors (BW, AL, KG, CV, MW and SB) had access to the dataset and accept final responsibility for the decision to submit for publication.

## Results

**Fig. 1:**
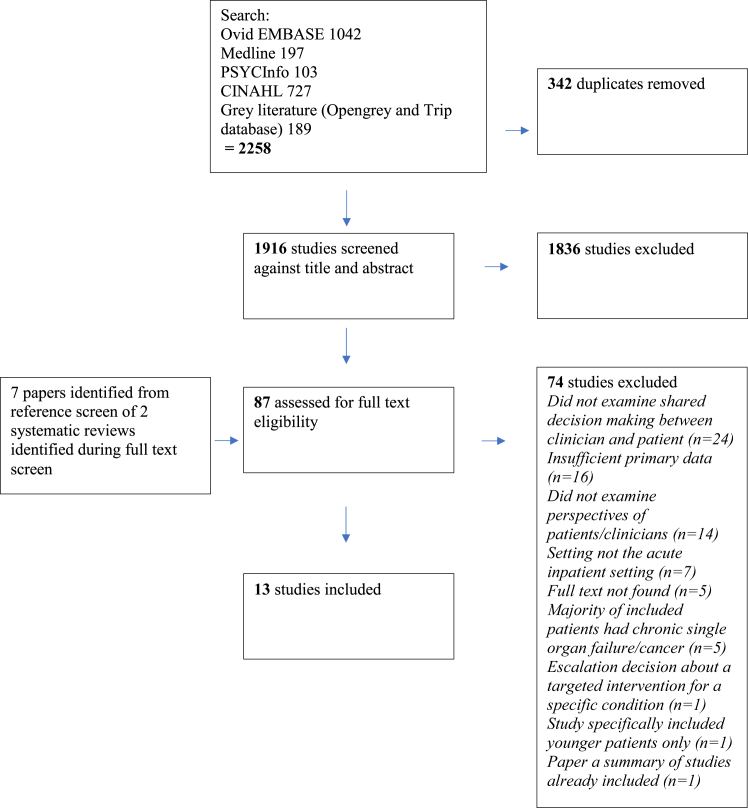
Preferred reporting items for systematic reviews and meta-analyses (PRISMA) flowchart. Flowchart illustrating systematic selection of papers for inclusion in the evidence synthesis.

Following duplicate exclusion, 1916 studies were identified from the initial search, 87 assessed for full text eligibility and 13 included in the synthesis (Fig. 1). The most common reasons for exclusion at full text review were that the studies did not examine shared decision-making between clinician and patient, did not examine patient or clinician perspectives or included insufficient primary data for synthesis.

The number of studies identified was considered appropriate for synthesis given the rich data, and is consistent with other thematic syntheses,^46^ therefore a decision was made to include all studies.

### Description of studies

There were 13 studies suitable for inclusion (Table 2), comprising 11 qualitative, one mixed methods and one quantitative, published between 2006 and 2022. There were five from the UK, four from North America and four from Europe. Two study groups accounted for six of the studies (^19^^,^^49^, ^50^, ^51^, ^52^^,^^58^). Three papers^50^, ^51^, ^52^ derived from the same data set and a fourth paper^55^ was a secondary analysis. Of the studies with qualitative components, six observed clinician/patient encounters and interviewed clinicians, one observed clinician/patient encounters and interviewed patients, one interviewed patients only, two interviewed clinicians only, two interviewed patients and clinicians. The survey study involved clinicians only.

### Quality

All included studies used appropriate design. The main methodological concerns related to reporting of reflexivity, consideration of data saturation and insufficient detail regarding selection of participants during recruitment (Appendix).

### Themes

Seven analytic themes are presented, of which four are clinician-orientated and three patient-orientated.

#### Clinician-orientated themes

##### Theme 1: treatment escalation is a medical decision

###### Clinicians consider TEPs a medical decision

A prevailing idea was that TEPs are decided by clinicians, with emphasis that patients could not demand treatments.^49^^,^^58^ Clinicians held the power about planning for and ultimately responding to deterioration.^53^

*“They can agree or disagree with me, and we can talk a little more about it, but they cannot choose something I will not give them”*

*(clinician quote, interview)*^58^

###### Clinicians decide whether the patient is a ‘candidate’ for treatment

Patient clinical factors informed clinicians' TEP decisions. Some described futility as an absolute.^49^ Patient co-morbidities, baseline functioning and age or ‘biological age’ contributed to the medical decision.^49^^,^^52^^,^^54^^,^^59^

*“If we’ve got a 95-year-old patient who’s bedridden and demented. Well, I’m not going to resuscitate him. If we’ve got an 80-year-old woman who rides her bicycle every day, who doesn’t have any associated pathologies, I’ll resuscitate her. And then there’s the whole gamut in between!”*

*(clinician quote, interview).*^54^

Several studies identified awareness of variability between decision-makers and centres on extent of escalation, although without clear causal patterns. Occasionally this led to clinician conflict or a multidisciplinary team (MDT) was convened to reach a decision.^53^^,^^54^^,^^59^ Mixed messages from different team members caused confusion and distrust.^52^

*“There are some people that would continue to resuscitate … and just don’t want patients to die. With the best will in the world they will decide to keep going … and I’m not one of them”*

*(clinician quote, interview).*^59^

###### Clinicians do not believe all acute inpatients need a TEP and make decisions about when it is discussed

Frequently, clinicians independently selected those patients they felt required TEP decisions.^49^, ^50^, ^51^ The focus of the conversation was also clinician-led and decisions about CPR usually prioritised.^50^^,^^57^

Clinicians often did not discuss TEP when they deemed full escalation to be appropriate because of reversible pathophysiology or where short admission was anticipated for a stable patient.^50^^,^^51^ One source commented that certain patient cohorts, such as those with iatrogenic complications or onco-haematological conditions, are more likely to be escalated.^53^

*“The pathology that had caused all of that derangement was expected to be quite reversible (…) it would make perfect sense to try and resuscitate her because there’s a good chance that we'd be able to”*

*(clinician quote, interview).*^51^

TEP was mostly discussed when patient deterioration was likely and escalation considered inappropriate because of underlying health problems.^49^ Sometimes a poor baseline made the escalation plan seem a foregone conclusion and clinicians did not feel an imperative to share their decision with the patient or colleagues,^54^ although others believed informing patients and families of the medical plan remained important.^49^

*“Elderly, demented, bedridden … No need to discuss with the family; in such easy cases, I make the decision all by myself”*

*(clinician quote, interview).*^54^

##### Theme 2: clinicians want the best for their patients amidst uncertainty

###### Clinicians try to make the best decision in uncertain circumstances

Clinicians considered TEPs important, complex and challenging.^54^ There was uncertainty about patients' trajectories and consequences of treatment.^49^, ^50^, ^51^^,^^59^ Information-gathering was important but challenged by meeting patients for the first time.^49^ There was pressure to make a decision in the ‘window’ between early deterioration and loss of capacity.^51^^,^^58^ Clinician experience was helpful^59^ but even experienced clinicians did not always achieve their anticipated clinical outcome.^55^

*‘Making these determinations was fraught with uncertainty. To manage this uncertainty, consultants relied on their predictions and imaginings of patients’ immediate futures'*

*(author comment).*^51^

Navigating clinical complexity with patients, especially around quality-focussed outcomes which some clinicians recognised to be subjective, was especially challenging.^19^^,^^52^^,^^53^^,^^58^^,^^59^

*“It is another dimension of choice, as it is not possible with factual knowledge to help people on their way to make the choice that is existentially best for them.”*

*(clinician quote, interview).*^58^

*‘It (the dilemma) usually concerned patients with advanced disease as these patients could benefit from life-sustaining interventions, but their long-term survival prognosis and their capacities for cognitive and functional recovery were limited.’*

*(author comment).*^53^

###### Clinicians are motivated by beneficence and non-maleficence

Clinicians pursued what they perceived to be the best survival outcome for the patient.^51^^,^^59^ Balancing clinical expertise with patient autonomy was difficult, and clinicians sometimes made ‘best interests’ decisions contrary to patient wishes.^55^

*‘The continuous framing of ceiling of treatment decisions around clinician-perceived patient benefit was a ubiquitous finding, and respondents almost universally stated early in the interviews that doing the best thing for the patient formed the basis of all subsequent decisions.’*

*(author comment).*^59^

A recurrent theme was that treatments can carry harm, especially in the elderly.^59^ When clinicians felt that limiting treatment was appropriate, they framed death as the natural course as opposed to ‘prolonging agony’.^49^^,^^55^ It was viewed as important for patients to be able to decline treatment.^58^

*“If I ever want to punish my worst enemy on the planet, I would make sure to get rid of all their family, put the person in a nursing home when they get really old, pump them full of drugs and then don't sign a DNR so that they get pricked with needles until they're 94 and basically a vegetable.”*

*(clinician quote, interview).*^55^

Treatment escalation was recognised to be a high-stakes decision and this weighed heavily on some doctors.^49^^,^^54^

*‘Some young physicians complained about the psychological burden associated with doubt, uncertainty, guilt, and regret after end-of-life decisions, which were described as “irreversible,” “life-or-death,” and “on-a-razor’s-edge” decisions: “Who I am to decide whether this person is to die today?”’*

*(author comment)*^54^

##### Theme 3: involving patients and families in decisions is not always meaningful and can involve conflict

Communication challenges were apparent throughout many of the themes but particular considerations are described below.

###### Shared decisions are important but difficult to navigate

Some clinicians sought to understand patients' values.^51^ Mostly this informed a wider decision-making process or the clinicians’ own subjective decision about a best interest decision.^59^ In exceptional cases clinicians felt obligated to enact a treatment plan decided by the patient^50^ or worked towards specific patient goals.^53^ Involvement in decision-making was believed to empower patients and some found patients appreciated TEP discussions.^19^ Patient involvement was most welcomed when making decisions around end of life.^50^

*“we’ve got their values and preferences fed into this discussion about what we might do in the event that things deteriorate”*

*(clinician quote, interview)*^51^

However, clinicians observed challenges in achieving meaningful patient involvement. Patients were viewed as emotional rather than rational.^58^ Concepts were complex, especially for unwell patients where it was difficult to navigate conveying sufficient but not excessive information.^49^^,^^50^^,^^59^ Clinicians did not always communicate complex ideas effectively.^55^ Decisions were often framed in terms of treatments.^57^

*“I didn’t want to overwhelm him, you know. (...) I wasn’t sure he was able to understand what ICU might have meant or all this sort of things”*

*(clinician quote, interview)*^50^

*“Would you want chest compressions, shocks to the heart, an artificial airway down the throat and potential life support?”*

*(clinician quote, observed).*^57^

Several clinicians remarked that their training did not prepare them for complex and emotional TEP conversations.^49^^,^^55^ Some conversations were therefore avoided or treatment perceived to be an easier course of action.^51^^,^^54^

*‘Residents … received very little training on code status discussions’*

*(author comment)*^55^

###### Clinicians seek to persuade towards a shared ‘correct’ decision

Clinicians largely felt obligated to inform patients and families of the TEP decision.^49^ Recurrently, rather than seeking discussion, most hoped to guide patients towards agreeing with the medical decision through persuasive conversations.^49^^,^^50^^,^^52^^,^^55^^,^^58^

*“I know it's terrible but you have in your mind what you think they should be (full code or DNR) and you talk them a certain way”*

*(clinician quote, interview)*^55^

Maintain trust and a good relationship despite potentially distressing conversations was important. Strategies included normalising the conversation,^55^ making it part of wider care,^51^ using a step-by-step approach^49^ and honesty.^49^ Some found a formalised process with a TEP form helpful.^58^ The *‘finality of medical decisions’*^49^ was perceived to be reassuring.

*“I can see these are really intense things. I can see you are sad. There is actually something we can do to make this easier. I have this document that also helps me to do this in a proper way, these difficult thoughts and feelings.”*

*(clinician quote, interview)*^58^

###### The TEP conversation can challenge the clinician–patient relationship

Clinicians recognised that TEP conversations could be distressing and feared a breakdown of trust.^49^^,^^52^

*“if you’re not careful with your language, a patient might interpret a discussion about what to do in the event of deterioration, escalation, CPR, et cetera, as you giving up on them, as you not being prepared to do everything that you can to get them over their illness”*

*(clinician quote, interview).*^49^

Anticipation of conflict was common, ideally avoided but sometimes inevitable.^49^^,^^50^ This could prevent a TEP decision being reached.^50^ Clinicians judged patients who disagreed with them to be challenging or not engaging in the process.^52^^,^^55^

*‘As we step out of the room, the resident exclaims, “She's delusional. She doesn't want to face reality!”’*

*(author field note)*^55^

###### Families are viewed as ‘sensible’ or ‘difficult’

The role of patients' families was widely discussed. When supportive of clinicians’ views, family involvement was considered constructive, but clinicians found disagreement with their professional opinion challenging.^49^^,^^54^

*“Clearly, the family has helped me. They were very cooperative”*

*(clinician quote, interview)*^54^

Clinicians often remarked that families did not understand treatment implications consequently demanded higher treatment escalation than clinicians felt appropriate.^55^^,^^60^ Families were believed to conflate treatment limitations with clinicians abandoning their loved ones.^53^^,^^54^

*“Some families demand everything, even though it is futile”*

*(clinician quote, interview).*^53^

Clinicians felt that capacitous patients should not be influenced by families but believed families should be aware of decisions. Sometimes, clinicians conceded to pressure from families and offered more treatment, but mostly they emphasised managing expectations and setting boundaries.^49^

*‘avoid conveying that medical decisions required relatives’ approval’*

*(author comment).*^49^

##### Theme 4: treatment escalation planning exists within the clinical environment, organisation and society

###### The hectic clinical environment with competing pressures influences how decisions are made

TEP occurred within a hectic clinical environment. Urgent pace and lack of privacy impaired complex, sensitive conversations.^49^ Clinicians sometimes struggled to balance immediate clinical tasks with TEP conversations.^52^^,^^57^^,^^60^

*“I’ve got a lot of patients to see, I, I try to be very patient-focussed and follow their agenda, but sometimes, I’ve gotta, I’ve gotta do what I’ve gotta do”*

*(clinician quote, interview)*^52^

It was widely felt that the conversation was better had by clinicians with existing relationship and rapport.^49^^,^^54^^,^^58^

###### TEP is influenced by organisational context

Organisational expectations could prompt TEP decision-making, or make the process feel ‘tick-box’;^50^, ^51^, ^52^ some organisations were viewed to lack a culture of involving patients.^58^ Intensive care resources were only occasionally cited but seen as potentially relevant.^59^ Some clinicians remarked a culture of DNAR decisions being synonymous with limitations on other aspects of care.^55^

Clinicians felt responsibility towards colleagues^50^ and hoped that timely TEP would protect against the distress of delivering aggressive and unhelpful treatments.^55^

*“if (the patient) were to deteriorate over the weekend he, you know, there’d be a much clearer plan for the on-call team”*

*(clinician quote, interview)*^50^

###### TEP is influenced by societal context

Clinicians reflected on a local societal view where the prospect of health deterioration was not yet normalised and patients did not instigate conversations; where they existed, community forms were valuable.^19^^,^^51^^,^^58^

*“This should preferably be founded in a culture where this is something you can talk about”*

*(clinician quote, interview)*.^19^

#### Patient-orientated themes

##### Theme 5: patients’ relationships with treatment escalation planning are complex

###### Patients value having a role in decisions about their health

Included patients were aware of deterioration and wanted a voice in the decision.^56^^,^^58^

*“I don’t want my wife or my husband saying put me on life support ….This is my decision..”*

*(patient quote, interview)*.^56^

###### Understanding of escalation comes from personal experience

Patients were informed by personal or observed experience of intensive treatments.^57^ Patients in one study expressed variable opinions about the chance of recovery they would be willing to accept.^56^

*“I’ve had friends of mine on life support. To me, they just turn out to be a vegetable there, waiting (…) I don’t think I would want it”*

*(patient quote, interview)*.^57^

###### A focus on hoping for the best

However, some patients expressed distress at the prospect of ill health or preferred to focus on the present.^19^^,^^50^^,^^58^ In one study, there was faith that an all-powerful God would ultimately decide each person's fate. When faced with poor prospects, patients hoped for a miracle.^56^

*“I think that people hang onto miracles (…) They are going to be the one in a gazillion that do wake up”*

*(patient quote, interview)*^56^

##### Theme 6: interactions with doctors are important but communication is not always easy

###### Patients put trust in doctors

Patients trusted doctors and were influenced by their opinions.^56^ They valued explanation of medical concepts.^19^

*“I know that I can look to (my doctor) and she wouldn’t be trying to pull the wool over my eyes. She would just give me the facts. That’s all there is to it ….I mean I would listen to her …”*

*(patient quote, interview)*.^56^

###### Patients and clinicians are not always on the same page when communicating about medical concepts

Patients did not always understand medical technicalities and therefore made requests or stated fears that doctors deemed illogical.^52^^,^^55^ Outcomes rather than treatments were important.^56^ Patients recognised lack of medical knowledge.^19^^,^^58^ Researchers commented that doctors did not always find effective strategies to communicate medical ideas,^52^^,^^55^ while patients could struggle to communicate their own perspectives.^57^

*‘the intern used euphemisms like ‘doing everything’, which her patient did not understand to include CPR’*

*(author comment)*^55^

##### Theme 7: patients are highly aware of their families when considering TEP

It was important for some patients to include families in conversations; others preferred to maintain autonomy, but still wanted families to be aware of their wishes. Formal TEP conversations helped ensure relatives understood their loved ones’ views.^19^^,^^49^

*“I am happy that my children now also know my wishes”*

*(patient quote, interview)*^19^

Patients wanted to spare families the burden of making difficult decisions or seeing them in distress. Families similarly wanted to prevent loved ones from suffering.^56^

*“There is no sense in putting hardship on my family by putting me on a machine and seeing me lay there on the machine”*

*(patient quote, interview)*.^56^

### Confidence in the review findings

Using the GRADE-CERQual approach, there were three findings with high confidence, three with moderate confidence and one with low confidence (Evidence profile, Table 3). There were widespread methodological concerns; the main reason to downgrade overall confidence was “adequacy” (richness or quantity of data).

**Table 3:** Evidence profile showing detailed assessment of confidence in the evidence synthesis findings.

	Studies contributing to finding	Methodological limitations	Coherence	Adequacy	Relevance	Overall CERQual assessment	Explanation of CERQual assessment
Theme 1: Treatment escalation is a medical decision	49, 50, 51, 52, 53, 54,57, 58, 59	Moderate concerns Several studies contributing to this finding lacked methodological detail on reflexivity (seven studies), saturation (seven studies), recruitment (four studies)	No or minor concerns	No or minor concerns	Minor concerns Five studies where all or most patients met age criteria, in the remainder age was not specified. Two studies focussed on scenarios where patients are often too unwell to engage in discussions but did include decisions where the patient could participate; one included in- and outpatient settings with participants envisaging acute setting decisions.	High confidence	
Theme 2: Clinicians want the best for their patients amidst uncertainty	19,49,51, 52, 53, 54, 55,58,59	Moderate concerns Several studies contributing to this finding lacked methodological detail on reflexivity (seven studies), saturation (six studies), recruitment (three studies)	No or minor concerns	No or minor concerns	Minor concerns Four studies where all or most patients met age criteria, in the remainder age was not specified. Two studies focussed on scenarios where patients are often too unwell to engage in discussions but did include decisions where the patient could participate; two included in- and outpatient settings with participants envisaging acute setting decisions.	High confidence	
Theme 3: Involving patients and families in decisions is not always meaningful and can involve conflict	19,49, 50, 51, 52, 53, 54, 55,57, 58, 59, 60	Moderate concerns Several studies contributing to this finding lacked methodological detail on reflexivity (nine studies), saturation (nine studies), recruitment (five studies)	No or minor concerns	No or minor concerns	Minor concerns Six studies where all or most patients met age criteria, in the remainder age was not specified. Two studies focussed on scenarios where patients are often too unwell to engage in discussions but did include decisions where the patient could participate; two included in- and outpatient settings with participants envisaging acute setting decisions.	High confidence	
Theme 4: Treatment escalation planning exists within the clinical environment, organisation and society	19,49, 50, 51, 52,54,55,57, 58, 59, 60	Moderate concerns Several studies contributing to this finding lacked methodological detail on reflexivity (eight studies), saturation (one study), recruitment (three studies)	Minor concerns Details on the nature of external influences raised by individual studies so not possible to examine specific influences such as resource availability, consequences of DNAR decisions on other treatments	No or minor concerns	Minor concerns Five studies where all or most patients met age criteria, in the remainder age was not specified. One study focussed on scenarios where patients are often too unwell to engage in discussions but did include decisions where the patient could participate; two included in- and outpatient settings with participants envisaging acute setting decisions.	Moderate confidence	Due to moderate concerns about methodology, minor concerns about coherence and minor concerns about relevance
Theme 5: Patients’ relationships with Treatment escalation planning are complex	19,50,56, 57, 58	Minor No discussion around reflexivity or saturation in any of the studies. Recruitment described in all.	Moderate Patient views on TEP often not clearly captured	Moderate Relatively small number of studies with variably rich and thin data	Minor Three studies where all or most patients met age criteria, two where age was not specified. Two of the studies took place in the acute setting, two included in- and outpatient settings with participants envisaging acute setting decisions, one included only participants envisaging the acute setting	Low confidence	Due to moderate concerns about coherence and adequacy with minor concerns about methodology and relevance
Theme 6: Interactions with doctors are important but communication is not always easy	19,52,55,56, 57, 58	Minor No discussion around reflexivity or saturation in any of the studies. Recruitment described in all but one.	Minor Communication challenges inferred by researchers observing in three studies so cannot be clear whether the data support the review finding	Minor Findings from six studies but data are not rich	Minor Four studies where all or most patients met age criteria, two where age was not specified. Three of the studies took place in the acute setting, two included in- and outpatient settings with participants envisaging acute setting decisions, one included only participants envisaging the acute setting	Moderate confidence	Due mainly to moderate concerns about adequacy, also moderate concerns about relevance and minor concerns about methodology and coherence
Theme 7: Patients are highly aware of their families when considering TEP	19,49,56	Minor concerns No discussion around reflexivity or saturation in any of the studies. Recruitment described in all.	Minor concerns Patients mostly wanted to involve families but in one study some participants wanted to make their own decisions	Moderate concerns Small number of studies contributing with limited data	Moderate concerns One studies where all or most patients met age criteria, two where age was not specified. One of the studies took place in the acute setting, another included in- and outpatient settings with participants envisaging acute setting decisions, one included only participants envisaging the acute setting	Moderate confidence	Due mainly to moderate concerns about adequacy, also moderate concerns about relevance and minor concerns about methodology and coherence

### Gaps in understanding

**Fig. 2:**
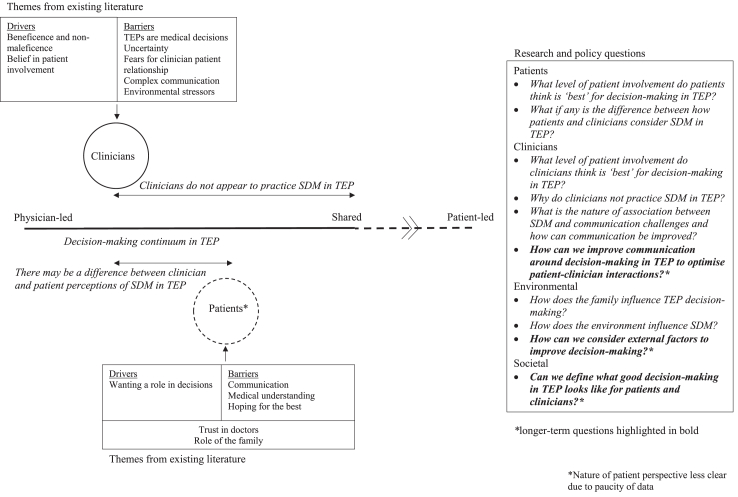
Existing literature on SDM in treatment escalation planning with suggestions for future research. Summary of existing literature on patient and clinician perspectives of shared decision-making for treatment escalation planning in the acute setting and suggestions for research. SDM = Shared decision-making; TEP = Treatment escalation planning.

We present a schematic demonstrating discrepancies between current clinician TEP decision-making approaches and SDM (Fig. 2). Patients views are less clear from the available evidence, but there may also be discordance between patients and clinicians regarding desired extent of patient involvement. Factors influencing TEP decision-making highlighted by existing literature include communication challenges, external factors and clinician emphasis on *beneficence* and *non-maleficence*.

## Discussion

This evidence synthesis found a clinician focus on medically-led TEP decisions motivated by achieving the best for patients and challenged by complex decisions, communication and environmental factors. There was a paucity of patient-focussed research but some evidence to demonstrate a willingness to engage, alongside communication barriers. This comprehensive synthesis is the first we are aware of exploring perceptions of SDM around TEP in the acute hospital setting.

Clinicians considered TEPs medical decisions. The doctor–patient relationship is evolving from a paternalistic model to more balanced partnership.^33^^,^^70^^,^^71^ Shared decision-making is increasingly an expectation for clinicians in the European and North American clinical settings contextualising the included studies. However, clinicians may see their role as decision-maker or believe patients do not wish to be involved.^25^^,^^31^^,^^72^ Clinicians represented in this synthesis appeared motivated by *beneficence* and *non-maleficence* but did not practice SDM, implying disconnect between academic- or policy-driven priorities and clinically-perceived appropriateness of SDM around TEPs in the acute setting.

In the small number of patient-focussed studies, there were disparate views captured on anticipation of ill-health and involvement in decision-making. Patients can struggle to identify values and priorities.^73^ Studies seeking to determine generalisable views have reached discordant conclusions, including comfort prioritisation,^74^ survival^75^ or variability,^76^ indicating ongoing importance of engaging the individual. Patient views as well as desire for involvement in decision-making may change.^77^^,^^78^ Goals of care and treatment preferences may differ,^79^ and although formalised TEPs may increase alignment^80^ discordance remains between clinician-documented plans and patient preferences.^81^, ^82^, ^83^, ^84^ In this synthesis, consistent with a recent review of decision-making around CPR in a UK-wide setting,^85^ patient perspectives on involvement in TEP decision-making remain poorly understood.

Communication was challenging for both patients and clinicians. Effective communication of complex medical concepts is difficult,^86^ especially with time contraints.^31^^,^^72^ Patients may not feel empowered to contribute,^31^ especially those who are older^77^ or have less ‘informational capacity’.^72^ Some fear distressing conversations.^72^ Patient and clinician perspectives on priorities for life sustaining treatment can differ^87^ and patients may make requests that healthcare professionals deem inappropriate.^88^ In the studies included, clinicians sought to avoid conflict whilst agreeing the medically-endorsed decision, while patients valued clinicians' opinions but could not always communicate or comprehend relevant information.

External factors also informed decision-making approaches. Organisational culture, resources, workflows and clinician-training influence SDM.^72^^,^^89^ The surrogate role in TEP is complex and important in several cultural contexts.^90^, ^91^, ^92^, ^93^ Although not our focus, family involvement was highlighted by both clinicians and patients: clinicians anticipated conflict about treatment limitations; patients were highly aware of their families but the nature of influence on decision-making was not clear. Clinicians were influenced by organisational expectations and environmental pressures, consistent with existing research.^94^ There was some reference to lack of societal awareness on planning for ill-health.

The main limitation is paucity of studies examining patient perspectives. Researchers may anticipate concerns about gaining ethical approval for studies involving patients in potentially distressing discussion around TEP, even though patients are often eager to share their experiences.^95^ Reflecting the importance of representing patients while acknowledging limited data, we include patient-orientated themes but with low and moderate confidence.

The weight of evidence derived from two research groups meaning our conclusions may be biased towards a narrower range of experience. The studies included all took place in European or North American settings, which may reflect different terminology around TEP not captured in our search strategy, limiting transferability to other settings. We recognise that our findings are unlikely to reflect approaches in settings with different expectations of the doctor–patient relationship or resource constraints. In keeping with many other qualitative studies, we decided only to include English language studies so that the interpretation and analysis by authors whose first language is English could reflect the nuance of direct participant quotations which might be lost through translation. Although we did not intend to focus on doctor-patient decision-making, the views of wider professional groups appear underrepresented in this literature.

There were methodological quality concerns in several included qualitative studies around adequate discussion of reflexivity, recruitment and data saturation. As is usual practice for thematic syntheses, we have used available data from primary studies, but note that these are selected and may be considered distinct from the original data.^47^^,^^96^

None of the studies identified were specifically designed to explore the ‘*shared’* element of decision making, which is the focus of this review. This may reflect a recent shift in emphasis whereby SDM is increasingly explored in emergency as well primary care settings.^33^^,^^34^ However, much of the data and analysis presented in these primary studies focussed on the interplay between clinician and patient and family views, and were thus adequate to address our research questions. Nonetheless, expectations for TEP decision-making continue to evolve, so the studies included may not represent most recent local practice.

We demonstrate with high confidence that clinician focus on medical decision-making is influenced by decision-making complexity and achievement of clinically-defined ‘good’ outcomes. TEP decisions are hypothetical and uniquely ‘high-stakes’; they require integration of complex physiological, psychological and ethical factors. Even so, these findings may be relevant to other settings involving complex decisions.

Furthermore, we identify a research priority to explore patient perceptions, for example through recruiting patients to qualitative interview or ethnographic studies focussed on SDM in TEP.

Specific focus is needed on the ‘shared’ element of SDM for TEP in the acute setting: can we define what ‘good’ decision-making in TEP looks like for older patients and clinicians? Given an ageing and increasingly co-morbid population with significant emergency care needs, these questions are widely relevant.

In conclusion, based on current evidence, TEP decisions appear dominated by clinicians' perspectives, motivated by achieving the best for patients and challenged by complex decisions, communication and environmental factors; patients’ perspectives have seldom been explored, but their input may be modest. Presenting the context and challenge of SDM during professional education may allow reflection and a more nuanced approach. Future research should seek to understand what approach to TEP decision-making patients and clinicians consider to be optimum in the acute setting so that a mutually acceptable standard can be defined in policy.

## Contributors

BW was responsible for initial analysis and drafting of the article. BW and AL performed systematic literature searches and quality assessment. BW and AL accessed and verified the data. All authors (BW, AL, KG, CV, MW, SB) contributed to drafting and gave final approval for the article to be submitted for publication.

## Data sharing statement

The primary studies included in this synthesis are widely available to academic audiences.

## Declaration of interests

All authors declare no competing interests.

## Appendix A Supplementary data

Appendix

ENTREQ checklist
