# Supplementary material for: Perspectives of healthcare professionals and older patients on shared decision-making for treatment escalation planning in the acute hospital setting: a systematic review and qualitative thematic synthesis
Source: eClinicalMedicine. 2023 Aug 10;62:102144. doi: 10.1016/j.eclinm.2023.102144 (PMC10425683; doi:10.1016/j.eclinm.2023.102144)
Supplement: Appendix [file mmc1.pdf]

APPENDIX

Table of Contents

***Example search strategies*......1**

***Quality Assessment*......5**

***Table of original data with source text*......10**

Example search strategies

Embase Classic+Embase <1947 to 2022 September 23>

1 exp evidence based emergency medicine/ or exp emergency medicine/ or exp emergency treatment/ or exp emergency ward/ or exp emergency health service/ or exp emergency patient/ or exp emergency care/ or exp emergency/ or exp emergency physician/ 656923
2 emergenc*.mp. [mp=title, abstract, heading word, drug trade name, original title, device manufacturer, drug manufacturer, device trade name, keyword heading word, floating subheading word, candidate term word] 816798
3 acute depart*.mp. [mp=title, abstract, heading word, drug trade name, original title, device manufacturer, drug manufacturer, device trade name, keyword heading word, floating subheading word, candidate term word] 32
4 acute medic*.mp. [mp=title, abstract, heading word, drug trade name, original title, device manufacturer, drug manufacturer, device trade name, keyword heading word, floating subheading word, candidate term word] 8055
5 Acute service.mp. 157
6 acute care.mp. [mp=title, abstract, heading word, drug trade name, original title, device manufacturer, drug manufacturer, device trade name, keyword heading word, floating subheading word, candidate term word] 37866
7 exp clinical decision making/ or exp decision making/ or exp medical decision making/ or exp shared decision making/ 442324
8 decision*.mp. [mp=title, abstract, heading word, drug trade name, original title, device manufacturer, drug manufacturer, device trade name, keyword heading word, floating subheading word, candidate term word] 886316
9 exp physician attitude/ or exp doctor patient relation/ or exp doctor patient relationship/ or exp interpersonal communication/ 832118
10 physician attitude*.mp. [mp=title, abstract, heading word, drug trade name, original title, device manufacturer, drug manufacturer, device trade name, keyword heading word, floating subheading word, candidate term word] 57196
11 doctor patient relation*.mp. [mp=title, abstract, heading word, drug trade name, original title, device manufacturer, drug manufacturer, device trade name, keyword heading word, floating subheading word, candidate term word] 126050
12 physician patient relation*.mp. [mp=title, abstract, heading word, drug trade name, original title, device manufacturer, drug manufacturer, device trade name, keyword heading word, floating subheading word, candidate term word] 4668
13 doctor patient communication.mp. [mp=title, abstract, heading word, drug trade name, original title, device manufacturer, drug manufacturer, device trade name, keyword heading word, floating subheading word, candidate term word] 2134
14 Physician patient communication.mp. [mp=title, abstract, heading word, drug trade name, original title, device manufacturer, drug manufacturer, device trade name, keyword heading word, floating subheading word, candidate term word] 1701
15 interpersonal communication.mp. [mp=title, abstract, heading word, drug trade name, original title, device manufacturer, drug manufacturer, device trade name, keyword heading word, floating subheading word, candidate term word] 186883

16 Attitude of Health Personnel.mp. [mp=title, abstract, heading word, drug trade name, original title, device manufacturer, drug manufacturer, device trade name, keyword heading word, floating subheading word, candidate term word] 856
17 treatment escalat*.mp. [mp=title, abstract, heading word, drug trade name, original title, device manufacturer, drug manufacturer, device trade name, keyword heading word, floating subheading word, candidate term word] 1367
18 (Recommended summary plan for emergency care and treatment).mp. [mp=title, abstract, heading word, drug trade name, original title, device manufacturer, drug manufacturer, device trade name, keyword heading word, floating subheading word, candidate term word] 26
19 ceiling of care.mp. 140
20 ceiling of treatment.mp. [mp=title, abstract, heading word, drug trade name, original title, device manufacturer, drug manufacturer, device trade name, keyword heading word, floating subheading word, candidate term word] 88
21 limit treatment*.mp. [mp=title, abstract, heading word, drug trade name, original title, device manufacturer, drug manufacturer, device trade name, keyword heading word, floating subheading word, candidate term word] 654
22 Treatment limit*.mp. [mp=title, abstract, heading word, drug trade name, original title, device manufacturer, drug manufacturer, device trade name, keyword heading word, floating subheading word, candidate term word] 2975
23 exp life sustaining treatment/ 3584
24 Physician order* for life sustaining treatment.mp. [mp=title, abstract, heading word, drug trade name, original title, device manufacturer, drug manufacturer, device trade name, keyword heading word, floating subheading word, candidate term word] 265
25 (Emergency care and treatment plan*).mp. [mp=title, abstract, heading word, drug trade name, original title, device manufacturer, drug manufacturer, device trade name, keyword heading word, floating subheading word, candidate term word] 785
26 Emergency care treatment plan*.mp. [mp=title, abstract, heading word, drug trade name, original title, device manufacturer, drug manufacturer, device trade name, keyword heading word, floating subheading word, candidate term word] 3
27 treatment escalation limitation plan*.mp. [mp=title, abstract, heading word, drug trade name, original title, device manufacturer, drug manufacturer, device trade name, keyword heading word, floating subheading word, candidate term word] 5
28 no escalation of treatment.mp. [mp=title, abstract, heading word, drug trade name, original title, device manufacturer, drug manufacturer, device trade name, keyword heading word, floating subheading word, candidate term word] 16
29 ward based ceiling.mp. 4
30 full escalation.mp. 24
31 1 or 2 or 3 or 4 or 5 or 6 1042981
32 7 or 8 or 9 or 10 or 11 or 12 or 13 or 14 or 15 or 16 1733742
33 17 or 18 or 19 or 20 or 21 or 22 or 23 or 24 or 25 or 26 or 27 or 28 or 29 or 30 9544
34 31 and 32 and 33 977

Ovid MEDLINE(R) ALL <1946 to September 23, 2022>

1 exp Emergencies/42718
2 emergency ward.mp. [mp=title, book title, abstract, original title, name of substance word, subject heading word, floating sub-heading word, keyword heading word, organism supplementary concept word, protocol supplementary concept word, rare disease supplementary concept word, unique identifier, synonyms] 1020
3 emergenc*.mp. [mp=title, book title, abstract, original title, name of substance word, subject heading word, floating sub-heading word, keyword heading word, organism supplementary concept word, protocol supplementary concept word, rare disease supplementary concept word, unique identifier, synonyms] 533230
4 Acute medic*.mp. [mp=title, book title, abstract, original title, name of substance word, subject heading word, floating sub-heading word, keyword heading word, organism supplementary concept word, protocol supplementary concept word, rare disease supplementary concept word, unique identifier, synonyms] 4793
5 Acute depart*.mp. [mp=title, book title, abstract, original title, name of substance word, subject heading word, floating sub-heading word, keyword heading word, organism supplementary concept word,

protocol supplementary concept word, rare disease supplementary concept word, unique identifier, synonyms] 12
6 Acute service.mp. [mp=title, book title, abstract, original title, name of substance word, subject heading word, floating sub-heading word, keyword heading word, organism supplementary concept word, protocol supplementary concept word, rare disease supplementary concept word, unique identifier, synonyms] 92
7 Acute care.mp. [mp=title, book title, abstract, original title, name of substance word, subject heading word, floating sub-heading word, keyword heading word, organism supplementary concept word, protocol supplementary concept word, rare disease supplementary concept word, unique identifier, synonyms] 26503
8 exp Decision Making/ 227011
9 exp Physician-Patient Relations/ or exp "Attitude of Health Personnel"/ 235549
10 decision*.mp. [mp=title, book title, abstract, original title, name of substance word, subject heading word, floating sub-heading word, keyword heading word, organism supplementary concept word, protocol supplementary concept word, rare disease supplementary concept word, unique identifier, synonyms] 549473
11 physician attitude*.mp. [mp=title, book title, abstract, original title, name of substance word, subject heading word, floating sub-heading word, keyword heading word, organism supplementary concept word, protocol supplementary concept word, rare disease supplementary concept word, unique identifier, synonyms] 871
12 doctor patient relation*.mp. [mp=title, book title, abstract, original title, name of substance word, subject heading word, floating sub-heading word, keyword heading word, organism supplementary concept word, protocol supplementary concept word, rare disease supplementary concept word, unique identifier, synonyms] 4488
13 Physician patient relation*.mp. [mp=title, book title, abstract, original title, name of substance word, subject heading word, floating sub-heading word, keyword heading word, organism supplementary concept word, protocol supplementary concept word, rare disease supplementary concept word, unique identifier, synonyms] 76979
14 Doctor patient communication.mp. [mp=title, book title, abstract, original title, name of substance word, subject heading word, floating sub-heading word, keyword heading word, organism supplementary concept word, protocol supplementary concept word, rare disease supplementary concept word, unique identifier, synonyms] 1492
15 Physician patient communication.mp. [mp=title, book title, abstract, original title, name of substance word, subject heading word, floating sub-heading word, keyword heading word, organism supplementary concept word, protocol supplementary concept word, rare disease supplementary concept word, unique identifier, synonyms] 1164
16 interpersonal communication.mp. [mp=title, book title, abstract, original title, name of substance word, subject heading word, floating sub-heading word, keyword heading word, organism supplementary concept word, protocol supplementary concept word, rare disease supplementary concept word, unique identifier, synonyms] 1883
17 Attitude of Health Personnel.mp. [mp=title, book title, abstract, original title, name of substance word, subject heading word, floating sub-heading word, keyword heading word, organism supplementary concept word, protocol supplementary concept word, rare disease supplementary concept word, unique identifier, synonyms] 130341
18 (Recommended summary plan for emergency care and treatment).mp. [mp=title, book title, abstract, original title, name of substance word, subject heading word, floating sub-heading word, keyword heading word, organism supplementary concept word, protocol supplementary concept word, rare disease supplementary concept word, unique identifier, synonyms] 13
19 Treatment escalat*.mp. [mp=title, book title, abstract, original title, name of substance word, subject heading word, floating sub-heading word, keyword heading word, organism supplementary concept word, protocol supplementary concept word, rare disease supplementary concept word, unique identifier, synonyms] 504
20 Ceiling of care.mp. [mp=title, book title, abstract, original title, name of substance word, subject heading word, floating sub-heading word, keyword heading word, organism supplementary concept word, protocol supplementary concept word, rare disease supplementary concept word, unique identifier, synonyms] 44
21 Ceiling of treatment.mp. [mp=title, book title, abstract, original title, name of substance word, subject heading word, floating sub-heading word, keyword heading word, organism supplementary concept word, protocol supplementary concept word, rare disease supplementary concept word, unique identifier, synonyms] 31

22 Limit treatment*.mp. [mp=title, book title, abstract, original title, name of substance word, subject heading word, floating sub-heading word, keyword heading word, organism supplementary concept word, protocol supplementary concept word, rare disease supplementary concept word, unique identifier, synonyms] 406

23 Treatment limit*.mp. [mp=title, book title, abstract, original title, name of substance word, subject heading word, floating sub-heading word, keyword heading word, organism supplementary concept word, protocol supplementary concept word, rare disease supplementary concept word, unique identifier, synonyms] 1732

24 life sustaining treatment.mp. 1804

25 Physician order* for life sustaining treatment.mp. 170

26 (Emergency care and treatment plan*).mp. [mp=title, book title, abstract, original title, name of substance word, subject heading word, floating sub-heading word, keyword heading word, organism supplementary concept word, protocol supplementary concept word, rare disease supplementary concept word, unique identifier, synonyms] 38

27 Emergency care treatment plan*.mp. [mp=title, book title, abstract, original title, name of substance word, subject heading word, floating sub-heading word, keyword heading word, organism supplementary concept word, protocol supplementary concept word, rare disease supplementary concept word, unique identifier, synonyms] 3

28 treatment escalation limitation plan.mp. [mp=title, book title, abstract, original title, name of substance word, subject heading word, floating sub-heading word, keyword heading word, organism supplementary concept word, protocol supplementary concept word, rare disease supplementary concept word, unique identifier, synonyms] 4

29 No escalation of treatment.mp. [mp=title, book title, abstract, original title, name of substance word, subject heading word, floating sub-heading word, keyword heading word, organism supplementary concept word, protocol supplementary concept word, rare disease supplementary concept word, unique identifier, synonyms] 9

30 Ward based ceiling.mp. [mp=title, book title, abstract, original title, name of substance word, subject heading word, floating sub-heading word, keyword heading word, organism supplementary concept word, protocol supplementary concept word, rare disease supplementary concept word, unique identifier, synonyms] 2

31 1 or 2 or 3 or 4 or 5 or 6 or 7 558228

32 8 or 9 or 10 or 11 or 12 or 13 or 14 or 15 or 16 or 17 870846

33 18 or 19 or 20 or 21 or 22 or 23 or 24 or 25 or 26 or 27 or 28 or 29 or 30 4478

34 31 and 32 and 33 166

Quality Assessment

	Was there a clear statement of the aims of the research?	Is a qualitative methodology appropriate?	Was the research design appropriate to address the aims of the research?	Was the recruitment strategy appropriate to the aims of the research?	Was the data collected in a way that addressed the research issue?	Has the relationship between researcher and participants been adequately considered?	Have ethical issues been taken into consideration?	Was the data analysis sufficiently rigorous?	Is there a clear statement of findings?	How valuable is the research?
Eli 2020	Yes	Yes<br>*Examines experiences*	Yes<br>*Semi-structured* interviews to explore process and experiences in addition to observation. T*opic guide based on research questions and literature*	Can't tell<br>*Range of views and clinical specialties purposively sampled; Unclear whether participants were approached or volunteered. No details on participants who declined to participate*	Can't tell<br><br>Appropriate setting and data collection method<br><br>*No discussion about data saturation*<br><br>*Consultations were observed prior to participant interview, but only interview findings were reported*	No<br>*Relationship between researcher and interviewed clinicians not considered. No reflexive statement or discussion of bias.*	Yes<br>*Approvals obtained. Measures taken to maximise transparency to patients; participant confidentiality respected. PPI involvement. However, limited information on consent process*	Yes<br>*Clear description of thematic analysis. Supporting quotations. However, no reflexivity*	Yes<br><br>*Explicit findings*<br><br>*Credibility via use of two coders and senior researchers to develop findings*<br><br>*Findings clearly respond to research question*	*Discussion of findings in relation to current policy and scientific literature. Implications considered*
Eli 2021	Yes	Yes<br>*Observations and interviews exploring 'why, when and how'*	Yes<br>*Combination of observation and interview added depth*	Can't tell<br>*Setting appears reasonable but no information on recruitment*	Yes<br>*Clear description and justification of methods. No reference to*	No<br>*Researchers treated as part of clinical team but no critical examination of*	Can't tell<br>*Approvals obtained. Some relevant ethical considerations*	Yes<br>*Detailed analysis with supporting quotations and tables*	Yes<br>*Reference to research question, triangulation through use of*	*Findings in policy context, transferability considered*

					interview topic guide, no discussion about data saturation	*this relationship.*<br><br>*No reflexive statement*	*noted, such as patient being given option for researcher not to be present. Brief discussion*	*displaying quantitative data in addition to thematic analysis. However, no reflexivity*	*observation and interviews*	
Eli 2022a	Yes	Yes<br>*Explores why*	Yes<br>*Combination of observation and interview added depth. Details not well described*	Can't tell<br>*Same participants as Eli 2021; secondary analysis*	Can't tell<br>*Secondary analysis of (Eli 2021) therefore data saturation not addressed Clear description and justification of methods. Topic guide described.*	No<br>*No reflexivity Strength is opportunistic analysis of absence of TEP conversation*	Can't tell<br>*Approvals obtained, consent described. No discussion of factors relating to secondary data analysis*	Yes<br>*Detailed description of analysis with supporting quotations. However, no reflexivity*	Yes<br>*Reference to research question, triangulation*	*Limited transferability recognised by authors and discussed. Findings considered in context of literature and practical implications explored.*
Eli 2022b	Yes	Yes<br>*Seeks to understand barriers to ReSPECT conversations*	Yes<br>*Fieldnote data and interviews create thick description of each case*	Can't tell<br>*Setting appears reasonable but no information on recruitment NB same study as Eli 2021 and Eli 2022a with different analysis focus, therefore methods described previously and appropriately referenced*	Yes<br>*Clear description and justification of methods. Topic guide mentioned but not described. Data saturation mentioned but not defined*	No<br>*No reflexivity Strength is opportunistic analysis of absence of TEP conversation*	Yes<br>*Approvals obtained. Appropriate anonymisation. Brief consideration of ethical considerations relating to ethnographic research*	Can't tell<br>*Supporting quotations but no detailed description of analysis methods. No reflexivity*	Yes<br>*Triangulation, reference to research question, findings discussed and refined by team*	*Practical consideration of how research can be used*

Escher 2021	Yes	Yes<br>*Exploring factors that influence ICU admission decision-making*	Yes<br>*Design seems appropriate although not justified Secondary analysis*	Yes<br>*Detailed explanation and rationale*	Yes<br>*Description of data collection methods. Topic guide pretested and provided in supplementary information. Saturation not discussed*	Can't tell<br>*Description of interviewer characteristics with relevance to study, not considered in terms of potential bias*	Can't tell<br>*Approvals obtained. No consideration of issues*	Can't tell<br>*Clear description of analysis process. Limited data to support findings*<br>*No reflexivity in analysis*	Yes	*Findings in context of literature, suggestions for further research*
Fassier 2016	Yes	Yes<br>*Explores perceptions and attitudes*	Yes<br>*Rational for qualitative research presented although not for chosen methods*	Yes<br>*Detailed explanation including those who refused to participate*	Yes<br>*Clear, detailed description of data collection process including some consideration of saturation*	Yes<br>*Researcher background considered; reflections on ethical considerations including role of the researcher during data collection discussed*	Yes<br>*Very detailed description of ethical considerations in appendix*	Yes<br>*Detailed description of analysis methods and sufficient supporting data presented. Reflexivity in analysis not specifically discussed*	Yes<br>*Credibility considered, findings related to research question*	*Findings considered in the context of existing literature and practical implications discussed*
Jenkins 2015	Yes	Yes	Yes<br>*Implied but not explicit justification for ethnographic methods*	No<br>*Not explored*	*Yes*<br>*Detailed description of methods, saturation mentioned*	No<br>*Researcher's own background not considered but immersive ethnographic approach described*	Yes<br>*Approvals obtained*<br>*Brief description of issues*	Yes<br>*Detailed account, contradictory data considered. Researcher influence not discussed*	Can't tell<br>*In-depth exploration of ideas but single statement of findings not found. Member validation used*	*Contributions to existing literature and practical implications considered*
Rodriquez 2006	Yes	Yes<br>*To explore beliefs*	*Yes*<br>*But not justified*	Yes<br>*Detailed description including people who*	Yes<br>*Data collection methods clearly explained.*	No<br>*No reflexivity*	Yes<br>*Approvals obtained, description of consent*	Can't tell<br>*Detailed description of analysis, supporting*	Yes<br>*Credibility considered*	*Findings considered in context of literature,*

				declined to participate	*Sample size explained but saturation not discussed*		*process. Considered participant capacity. Potential ethical issues not discussed*	*data presented. However, methods labelled as grounded theory but approach described not consistent. Reflexive researcher role in analysis not considered.*		*transferability considered*
Shah 2017	Yes	Yes<br><br>*Explores barriers and Facilitators to goals of care discussions*	Yes<br>*Although not justified*	Can't tell<br>*Clear description of eligibility but unclear how participants were approached and any who declined*	Yes<br>*Data collection clearly described. Saturation not discussed*	No<br>*Not discussed*	Can't tell<br>*Approvals obtained. Potential issues including use of 'sham questions' to blind participants to the 'true nature of the study' not discussed*	Yes<br>*Analysis approach described. Researcher reflexivity not considered*	Yes<br>*Clear statement, credibility considered*	*Contribution to clinical practice. Limited discussion in context of literature but transferability considered*
Tuesen 2022a	Yes	Yes<br>*Explores perspectives*	Yes<br>*Although not justified*	Can't tell<br>*Recruitment described, although rationale for selection of specific patients not explained*	Yes<br>*Data collection clearly explained but saturation not discussed*	No<br>*No critical examination of researcher role, although study modifications due to COVID-19 described*	Can't tell<br>*Approvals obtained. No consideration of issues*	Yes<br>*Analysis described, supporting data presented. No reflexivity in analysis*	Can't tell<br>*Clear findings, links to research question, credibility not discussed*	*Findings in context of literature, limitations and transferability considered*
Tuesen *2022b	Yes	Yes<br><br>*As part of a mixed methods study*	Yes<br>*Although not justified*	Can't tell<br>*Describes sampling approach but limited detail*	Yes<br>*Clearly described Sample size justified but*	No<br>*Not discussed*	Can't tell<br>*Approvals obtained. No consideration of issues*	Yes<br>*Analysis described, supporting data presented.*	Can't tell<br>*Clear findings linked to research question.*	*Findings in context of literature, limitations and*

				on recruitment for interviews	*saturation not discussed*			*No reflexivity in analysis*	*Credibility not discussed*	*transferability considered*
Walzl 2019	Yes	Yes	Yes *Phenomenological approach discussed*	Yes	Yes<br><br>Detailed description including topic guide and saturation	Can't tell *Reflexive journals although no specific statement of reflexivity*	Yes *Approvals obtained, explanation to participants mentioned. However, potential issues not discussed*	Yes *Description of analysis process, sufficient supporting data, contradictory data considered. Reflexivity not discussed*	Yes *Findings reflect research question and presented in a model. Credibility discussed in detail*	*Contribution to understanding considered with recommendations for future research. Transferability considered. Limited reference to the literature.*

Table of original data with source text

Data	Source
"They can agree or disagree with me, and we can talk a little more about it, but they cannot choose something I will not give them" (clinician quote, interview)	1
"If we've got a 95-year-old patient who's bedridden and demented. Well, I'm not going to resuscitate him. If we've got an 80-year-old woman who rides her bicycle every day, who doesn't have any associated pathologies, I'll resuscitate her. And then there's the whole gamut in between!" (clinician quote, interview).	2
"There are some people that would continue to resuscitate…and just don't want patients to die. With the best will in the world they will decide to keep going… and I'm not one of them" (clinician quote, interview).	3
"The pathology that had caused all of that derangement was expected to be quite reversible (…) it would make perfect sense to try and resuscitate her because there's a good chance that we'd be able to" (clinician quote, interview).	4
"Elderly, demented, bedridden... No need to discuss with the family; in such easy cases, I make the decision all by myself"(clinician quote, interview).	2
'Making these determinations was fraught with uncertainty. To manage this uncertainty, consultants relied on their predictions and imaginings of patients' immediate futures' (author comment).	4
"It is another dimension of choice, as it is not possible with factual knowledge to help people on their way to make the choice that is existentially best for them." (clinician quote, interview).	1
'It (the dilemma) usually concerned patients with advanced disease as these patients could benefit from life- sustaining interventions, but their long- term survival prognosis and their capacities for cognitive and functional recovery were limited.' (author comment).	5
'The continuous framing of ceiling of treatment decisions around clinician-perceived patient benefit was a ubiquitous finding, and respondents almost universally stated early in the interviews that doing the best thing for the patient formed the basis of all subsequent decisions.' (author comment)	3
"If I ever want to punish my worst enemy on the planet, I would make sure to get rid of all their family, put the person in a nursing home when they get really old, pump them full of drugs and then don't sign a DNR so that they get pricked with needles until they're 94 and basically a vegetable." (clinician quote, interview).	6
'Some young physicians complained about the psychological burden associated with doubt, uncertainty, guilt, and regret after end-of-life decisions, which were described as "irreversible," "life-or-death," and "on-a-razor's-edge" decisions: "Who I am to decide whether this person is to die today?"'(author comment)	2
"we've got their values and preferences fed into this discussion about what we might do in the event that things deteriorate" (clinician quote, interview)	4

"I didn't want to overwhelm him, you know. (. . .) I wasn't sure he was able to understand what ICU might have meant or all this sort of things" (clinician quote, interview)	*7*
"Would you want chest compressions, shocks to the heart, an artificial airway down the throat and potential life support?" (clinician quote, observed).	*8*
'Residents … received very little training on code status discussions' (author comment)	*6*
"I know it's terrible but you have in your mind what you think they should be (full code or DNR) and you talk them a certain way" (clinician quote, interview)	*6*
"I can see these are really intense things. I can see you are sad. There is actually something we can do to make this easier. I have this document that also helps me to do this in a proper way, these difficult thoughts and feelings." (clinician quote, interview)	*1*
"if you're not careful with your language, a patient might interpret a discussion about what to do in the event of deterioration, escalation, CPR, et cetera, as you giving up on them, as you not being prepared to do everything that you can to get them over their illness" (clinician quote, interview).	*9*
'As we step out of the room, the resident exclaims, "She's delusional. She doesn't want to face reality!"' (author field note)	*6*
"Some families demand everything, even though it is futile" (clinician quote, interview).	*5*
'avoid conveying that medical decisions required relatives' approval' (author comment).	*9*
"I've got a lot of patients to see, I, I try to be very patient-focussed and follow their agenda, but sometimes, I've gotta, I've gotta do what I've gotta do" (clinician quote, interview)	*10*
"if (the patient) were to deteriorate over the weekend he, you know, there'd be a much clearer plan for the on-call team" (clinician quote, interview)	*7*
"This should preferably be founded in a culture where this is something you can talk about" (clinician quote, interview).	*11*
"I don't want my wife or my husband saying put me on life support....This is my decision.." (patient quote, interview).	*12*
"I've had friends of mine on life support. To me, they just turn out to be a vegetable there, waiting (…) I don't think I would want it" (patient quote, interview).	*8*
"I think that people hang onto miracles (…) They are going to be the one in a gazillion that do wake up" (patient quote, interview)	*12*
"I know that I can look to (my doctor) and she wouldn't be trying to pull the wool over my eyes. She would just give me the facts. That's all there is to it....I mean I would listen to her..." (patient quote, interview).	*12*
'the intern used euphemisms like 'doing everything', which her patient did not understand to include CPR' (author comment)	*6*
"I am happy that my children now also know my wishes"(patient quote, interview)	*11*
"There is no sense in putting hardship on my family by putting me on a machine and seeing me lay there on the machine" (patient quote, interview).	12

1. Tuesen LD, Ågård AS, Bülow H-H, et al. Decision-making conversations for life-sustaining treatment with seriously ill patients using a Danish version of the US POLST: a qualitative study of patient and physician experiences. *Scandinavian Journal of Primary Health Care* 2022; 40: 57-66. DOI: 10.1080/02813432.2022.2036481.
2. Fassier T, Valour E, Colin C, et al. Who am I to decide whether this person is to die today? Physicians' life-or-death decisions for elderly critically ill patients at the emergency department–ICU interface: a qualitative study. *Annals of emergency medicine* 2016; 68: 28-39.
3. Walzl N, Jameson J, Kinsella J, et al. Ceilings of treatment: A qualitative study in the emergency department. *BMC Emergency Medicine* 2019; 19: 9. DOI: https://dx.doi.org/10.1186/s12873-019-0225-6.
4. Eli K, Hawkes C, Perkins GD, et al. Caring in the silences: why physicians and surgeons do not discuss emergency care and treatment planning with their patients - an analysis of hospital-based ethnographic case studies in England.. *BMJ open* 2022; 12: e046189. DOI: https://dx.doi.org/10.1136/bmjopen-2020-046189.
5. Escher M, Nendaz MR, Cullati S, et al. Physicians' perspective on potentially non-beneficial treatment when assessing patients with advanced disease for ICU admission: A qualitative study. *BMJ Open* 2021; 11: e046268. DOI: https://dx.doi.org/10.1136/bmjopen-2020-046268.
6. Jenkins TM. 'It's time she stopped torturing herself': Structural constraints to decision-making about life-sustaining treatment by medical trainees. *Social Science and Medicine* 2015; 132: 132-140. DOI: https://dx.doi.org/10.1016/j.socscimed.2015.03.039.
7. Eli K, Hawkes CA, Ochieng C, et al. Why, when and how do secondary-care clinicians have emergency care and treatment planning conversations? Qualitative findings from the ReSPECT Evaluation study.. *Resuscitation* 2021; 162: 343-350. DOI: https://dx.doi.org/10.1016/j.resuscitation.2021.01.013.
8. Shah K, Swinton M and You JJ. Barriers and facilitators for goals of care discussions between residents and hospitalised patients. *Postgraduate medical journal* 2017; 93: 127-132. DOI: https://dx.doi.org/10.1136/postgradmedj-2016-133951.
9. Eli K, Ochieng C, Hawkes C, et al. Secondary care consultant clinicians' experiences of conducting emergency care and treatment planning conversations in England: an interview-based analysis. *BMJ open* 2020; 10: e031633. DOI: https://dx.doi.org/10.1136/bmjopen-2019-031633.
10. Eli K, Huxley CJ, Hawkes CA, et al. Why are some ReSPECT conversations left incomplete? A qualitative case study analysis.. *Resuscitation Plus* 2022; 10: 100255-100255. DOI: 10.1016/j.resplu.2022.100255.
11. Tuesen LD, Bülow H-H, Ågård AS, et al. Discussing patient preferences for levels of life-sustaining treatment: development and pilot testing of a Danish POLST form. *BMC Palliative Care* 2022; 21: 1-8. DOI: 10.1186/s12904-021-00892-2.
12. Rodriguez KL and Young AJ. Elderly veterans' beliefs concerning life-sustaining treatment and the control of their end-of-life health and health care. *Journal of Aging & Health* 2006; 18: 686-706. DOI: 10.1177/0898264306293258.
