# Supplementary material for: Perspectives of healthcare professionals and older patients on shared decision-making for treatment escalation planning in the acute hospital setting: a systematic review and qualitative thematic synthesis
Source: eClinicalMedicine. 2023 Aug 10;62:102144. doi: 10.1016/j.eclinm.2023.102144 (PMC10425683; doi:10.1016/j.eclinm.2023.102144)
Supplement: ENTREQ checklist [file mmc2.docx]

| Item | Included |
| --- | --- |
| Aim | Y |
| Synthesis methodology | Y |
| Approach to searching | Y |
| Inclusion criteria | Y |
| Data sources | Y |
| Electronic search strategy | Y |
| Study screening methods | Y |
| Study characteristics | Y |
| Study selection results | Y |
| Rationale for appraisal | Y |
| Appraisal items | Y |
| Appraisal process | Y |
| Appraisal results | Y |
| Data extraction | Y |
| Software | Y |
| Number of reviewers | Y |
| Coding | Y |
| Study comparison | Y |
| Derivation of themes | Y |
| Quotations | Y |
| Synthesis output | Y |
